# Comprehensive landscape of neutralizing antibody and cell-mediated response elicited by the 1/5 fractional dose of 17DD-YF primary vaccination in adults

**DOI:** 10.1038/s41598-024-57645-3

**Published:** 2024-04-02

**Authors:** Laise Rodrigues Reis, Ismael Artur Costa-Rocha, Thais Abdala-Torres, Ana Carolina Campi-Azevedo, Vanessa Peruhype-Magalhães, Márcio Sobreira Silva Araújo, Elaine Spezialli, Lis Ribeiro do Valle Antonelli, Rosiane Aparecida da Silva-Pereira, Gregório Guilherme Almeida, Eder Gatti Fernandes, Francieli Fontana Sutile Tardetti Fantinato, Carla Magda Allan Santos Domingues, Maria Cristina Ferreira Lemos, Alexandre Chieppe, Jandira Aparecida Campos Lemos, Jordana Grazziela Coelho-dos-Reis, Sheila Maria Barbosa de Lima, Adriana de Souza Azevedo, Waleska Dias Schwarcz, Luiz Antônio Bastos Camacho, Maria de Lourdes de Sousa Maia, Tatiana Guimarães de Noronha, Caroline Duault, Yael Rosenberg-Hasson, Andréa Teixeira-Carvalho, Holden Terry Maecker, Olindo Assis Martins-Filho, Collaborative Group for Studies of Yellow Fever Vaccine, Dayane Andriotti Otta, Olindo Assis Martins-Filho

**Affiliations:** 1 Instituto René Rachou, Fundação Oswaldo Cruz – FIOCRUZ-Minas, Belo Horizonte, MG Brazil; 2 Departamento de Bioquímica e Imunologia, Instituto de Ciências Biológicas, Universidade Federal de Minas Gerais, Belo Horizonte, MG Brazil; 3 Instituto de Infectologia Emílio Ribas, São Paulo, SP Brazil; 4 Departamento de Vigilância das Doenças Transmissíveis, Secretaria de Vigilância em Saúde, Ministério da Saúde, Brasília, DF Brazil; 5 External Consultant, Independent Researcher, Brasília, DF Brazil; 6 Secretaria de Estado de Saúde, Rio de Janeiro, RJ Brazil; 7 Superintendência de Vigilância em Saúde, Secretaria Municipal de Saúde do Rio de Janeiro, Rio de Janeiro, RJ Brazil; 8 Secretaria Municipal de Saúde de Belo Horizonte, Belo Horizonte, MG Brazil; 9 Laboratório de Virologia Básica e Aplicada, Instituto de Ciências Biológicas da Universidade Federal de Minas Gerais - UFMG, Belo Horizonte, MG Brazil; 10 Departamento de Desenvolvimento Experimental e Pré-Clínico, Instituto de Tecnologia em Imunobiológicos Bio-Manguinhos - FIOCRUZ, Rio de Janeiro, RJ Brazil; 11 Laboratório de Análise Imunomolecular, Instituto de Tecnologia em Imunobiológicos Bio-Manguinhos - FIOCRUZ, Rio de Janeiro, RJ Brazil; 12 Escola Nacional de Saúde Pública - FIOCRUZ, Rio de Janeiro, RJ Brazil; 13 Assessoria Clínica, Instituto de Tecnologia em Imunobiológicos Bio-Manguinhos - FIOCRUZ, Rio de Janeiro, RJ Brazil; 14 Human Immune Monitoring Center, Stanford University, Stanford, CA USA; 15 Department of Microbiology and Immunology, Stanford University, Stanford, USA; 16 Grupo Integrado de Pesquisas em Biomarcadores, Instituto René Rachou, Fundação Oswaldo Cruz – FIOCRUZ-Minas, Belo Horizonte, MG Brazil

**Keywords:** Yellow fever, 17DD vaccine, Fractional dose, Neutralizing antibodies, Cellular memory, Adults, Adaptive immunity, Chemokines, Cytokines, Lymphocytes, Vaccines, Live attenuated vaccines, Biomarkers

## Abstract

The present study aimed at evaluating the YF-specific neutralizing antibody profile besides a multiparametric analysis of phenotypic/functional features of cell-mediated response elicited by the 1/5 fractional dose of 17DD-YF vaccine, administered as a single subcutaneous injection. The immunological parameters of each volunteer was monitored at two time points, referred as: before (Day 0) [Non-Vaccinated, NV_(D0)_] and after vaccination (Day 30–45) [Primary Vaccinees, PV_(D30–45)_]. Data demonstrated high levels of neutralizing antibodies for PV_(D30–45)_ leading to a seropositivity rate of 93%. A broad increase of systemic soluble mediators with a mixed profile was also observed for PV_(D30–45)_, with IFN-γ and TNF-α presenting the highest baseline fold changes. Integrative network mapping of soluble mediators showed increased correlation numbers in PV_(D30–45)_ as compared to NV_(D0)_ (532*vs*398). Moreover, PV_(D30–45)_ exhibited increased levels of Terminal Effector (CD45RA^+^CCR7^−^) CD4^+^ and CD8^+^ T-cells and Non-Classical memory B-cells (IgD^+^CD27^+^). Dimensionality reduction of Mass Cytometry data further support these findings. A polyfunctional cytokine profile (TNF-α/IFN-γ/IL-10/IL-17/IL-2) of T and B-cells was observed upon in vitro antigen recall. Mapping and kinetics timeline of soluble mediator signatures for PV_(D30–45)_ further confirmed the polyfunctional profile upon long-term in vitro culture, mediated by increased levels of IFN-γ and TNF-α along with decreased production of IL-10. These findings suggest novel insights of correlates of protection elicited by the 1/5 fractional dose of 17DD-YF vaccine.

## Introduction

Yellow Fever (YF) is an acute viral disease caused by an RNA arbovirus (genus Flavivirus), endemic in several tropical and subtropical areas in Africa, Central and South America^1–3^. There are an estimated 80,000 to 200,000 cases of YF per year worldwide with mortality rate ranging from 20 to 60%^2^.

Considering that no specific treatment is available for YF, the primary measure for prevention and disease control is the live-attenuated YF vaccine available since 1937. The YF vaccine is among the most outstanding human vaccines ever developed, inducing long-lasting protective immunity in 95–99% adults upon primary vaccination^2,4^.

Despite the global efforts to eliminate YF epidemics, re-emergence of YF has been reported since 2016 mainly due to a progressive decrease in the vaccination coverage in endemic regions. Relevant outbreaks were reported in Angola, Democratic Republic of the Congo (2016), Brazil (2016–2017 and 2017–2018) and Guinea and Senegal (2020–2021)^5–8^. Large-scale YF vaccination campaigns have been employed to control these outbreaks, increasing the demand for doses and leading to a global shortage of international stockpile of YF vaccine. Aiming at ensuring an appropriate and coordinated distribution of limited vaccine stocks during outbreaks, the World Health Organization (WHO) has recommended the use of fractional dose of YF vaccine, as a dose-sparing option, to prevent YF spreading^9,10^. Under guidance from the WHO, the use of 1/5 of a standard dose of the Bio-Manguinhos vaccine (0.1 mL) has been recommended for large-scale campaigns in response to emergency situations.

Previous reports have provided strong evidence to support the use of fractional dose regimens as a viable approach for providing immunity throughout high seroconversion rates upon primary vaccination^11^. However, the immune response to fractional dose vaccination is not fully understood. The use of fractional dosing has been fundamentally supported by the quantification of YF-specific neutralizing antibodies, but data evaluating vaccine-induced cellular immunity using this regimen are still required. In the present investigation, we aimed at evaluating the magnitude of neutralizing antibody profile besides a multiparametric analysis of soluble mediators and their correlation with phenotypic/functional features of cell-mediated response. Our findings bring about novel insights regarding the correlates of protection, providing a comprehensive landscape of the immune response elicited by the 1/5 fractional dose of 17DD-YF vaccine.

## Results

### Neutralizing antibody titers and seropositivity rates in adults following 17DD-YF 1/5 fractional dose primary vaccination

**Figure 1:**
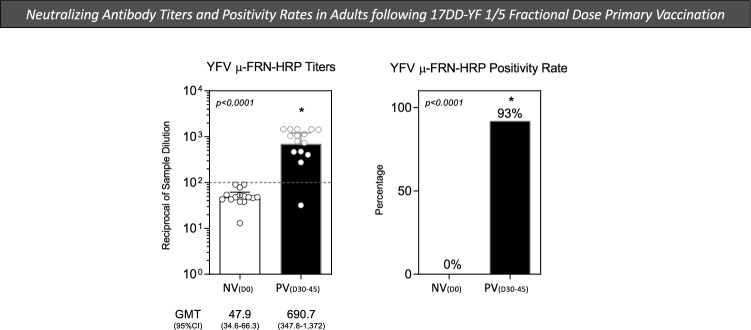
Neutralizing antibody titers and seropositivity rates in adults following 17DD-YF 1/5 fractional dose primary vaccination. The YF-specific neutralizing antibody titers were measured in heparin-free plasma samples from all volunteers before [NV_(D0)_ = ▭, n = 15] and 30–45 days after [PV_(D30–45)_ = ▬, n = 15] 17DD-YF 1/5 fractional dose primary vaccination. The micro Focus Reduction Neutralization—Horseradish Peroxidase (μFRN-HRP) was carried out as described in “Methods” section. Data are presented as scattering distribution of individual values (reciprocal of sample dilution) over bars representing the geometric mean titers with confidence interval (GMT, 95% CI). The positivity rate was calculated as the frequency (%) of subjects with titers higher than 1:100 (dashed line). Comparative analysis of geometric mean titers and positivity rates between NV_(D0)_ and PV_(D30–45)_ was carried out by Student t test and Chi-square test, respectively. In all cases, significant differences were considered at p < 0.05 and represented by *.

Evaluation of vaccine immunogenicity by using surrogate biological markers. In this context, the quantification of YF-specific neutralizing antibodies has been considered the gold standard. In the present study, the micro Focus Reduction Neutralization—Horseradish Peroxidase was used to quantify the YF-specific neutralizing antibodies titers and seropositivity rates, according to the standard protocol of μFRN-HRP described by Simões^12^. The results are shown in Fig. 1. Data analysis demonstrated that primary vaccination with 17DD-YF 1/5 fractional dose induced high levels of YF-specific neutralizing antibodies as observed for PV_(D30–45)_ (GMT = 690.7; 95% CI 347.8–1.372) with a seropositivity rate of 93% (Fig. 1). These findings demonstrated that 1/5 fractional dose of the 17DD-YF vaccine induces significant seroprotection in primary adult vaccinees.

### Changes in systemic soluble mediators in adults following 17DD-YF 1/5 fractional dose primary vaccination

**Figure 2:**
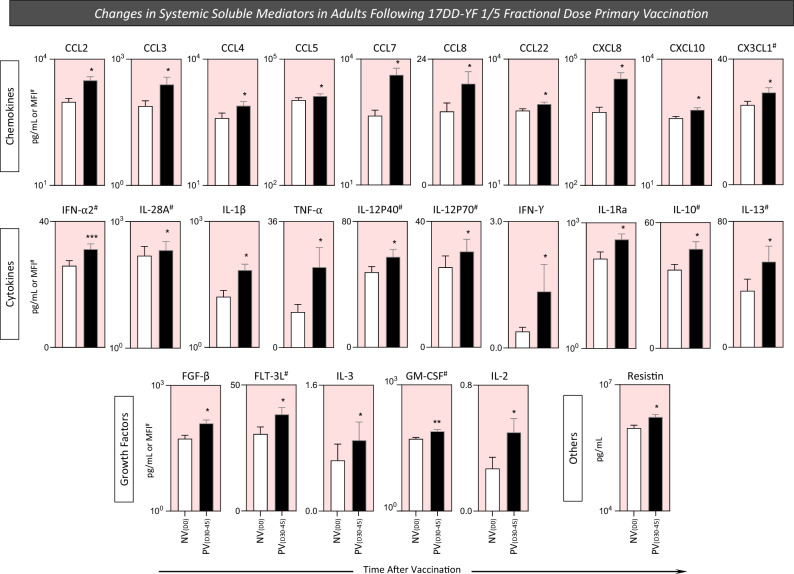
Changes in systemic soluble mediators in adults following 17DD-YF 1/5 fractional dose primary vaccination. Systemic soluble mediators were measured in plasma samples from all volunteers before [NV_(D0)_ = ▭, n = 15] and 30–45 days after [PV_(D30–45)_ = ▬, n = 15] 17DD-YF 1/5 fractional dose primary vaccination. Measurements were carried out by xMAP technology as described in “Methods” section. The results are shown as mean values ± standard error of plasma concentration expressed in pg/mL or MFI (#). Comparative analysis between NV_(D0)_ and PV_(D30–45)_ was carried out by Student t test. Significant differences at p < 0.05 are underscored according to p value (*p < 0.05; **p ≤ 0.01, ***p ≤ 0. 001). Pink background underscores the soluble mediators with increased levels in PV_(D30–45)_ as compared to NV_(D0)_.

The analysis of soluble immune mediators in plasma samples is a rational strategy to characterize the systemic impact of YF-vaccine in the innate and adaptive immune responses. Aiming at quantifying the systemic profile of soluble mediators before (D0) and after (D30-45) 17DD-YF 1/5 fractional dose primary vaccination, a 76 parameters Luminex xMAP technology was employed and the results are shown in Fig. 2. The primary vaccination with 17DD-YF 1/5 fractional dose elicited a broad increase of systemic soluble mediators with a mixed profile comprised of higher levels of chemokines (CCL2, CCL3, CCL4, CCL5, CCL7, CCL8, CCL22, CXCL8, CXCL10 and CX3CL1), pro-inflammatory cytokines (IFN-α2, IL-28A, IL-1β, TNF-α, IL-12P40, IL-12P70, IFN-α), regulatory cytokines (IL-1Ra, IL-10 and IL-13) and growth factors (FGF-β, FLT-3L, IL-3, GM-CSF and IL-2) and others (Resistin) observed for PV_(D30–45)_ as compared to NV_(D0)_ (Fig. 2). A range of systemic soluble mediators remains unaltered in PV_(D30–45)_ as compared to NV_(D0)_ (Supplementary Fig. 2). Our results demonstrated that a mixed profile, including regulatory and pro-inflammatory cytokines, was elicited by 17DD-YF 1/5 fractional dose primary vaccination, which substantiate the development of a robust immune response.

### Baseline fold changes in systemic soluble mediators in adults following 17DD-YF 1/5 fractional dose primary vaccination

**Figure 3:**
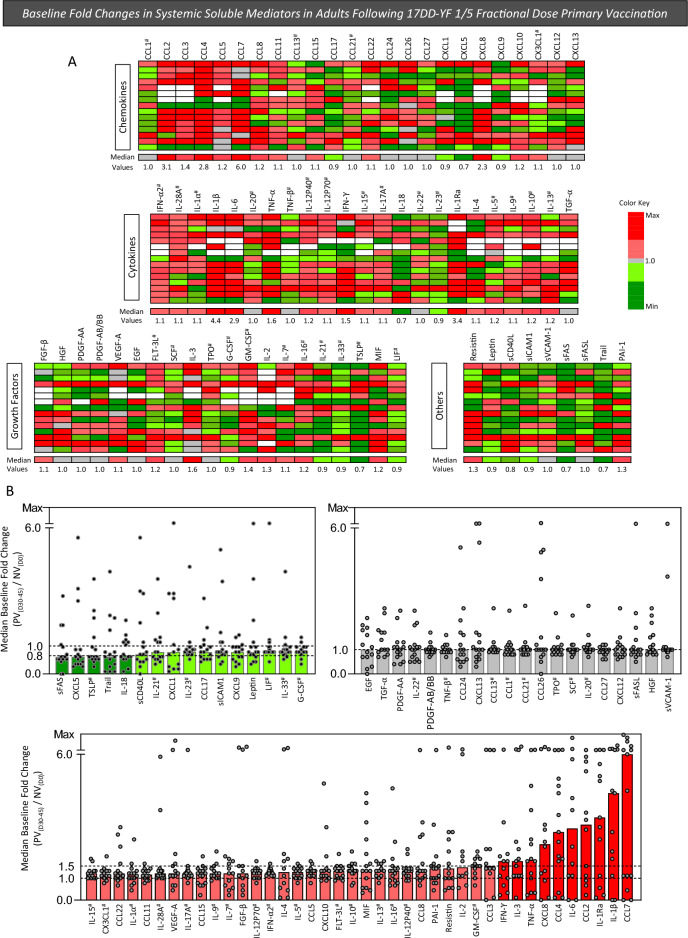
Baseline fold changes in systemic soluble mediators in adults following 17DD-YF 1/5 fractional dose primary vaccination. Systemic soluble mediators were measured in plasma samples from all volunteers before [NV_(D0)_, n = 15] and 30–45 days after [PV_(D30–45)_, n = 15] 17DD-YF 1/5 fractional dose primary vaccination. Measurements were carried out by xMAP technology as described in “Methods” section. The baseline fold change in the levels of each soluble mediator was calculated for individual samples as the ratio between values, expressed in pg/mL or MFI (#), observed at PV_(D30–45)_ divided by those detected at NV_(D0)_. The baseline fold change values were highlighted by shades of green to represent decreased (≥ 0.5 and ≤ 0.80/ or > 0.80 and < 1/) and red to underscore increased values (> 1 and ≤ 1.50/ or > 1.50/) baseline fold change values. Unaltered levels (baseline fold change = 1.0) are represented by gray color () and missing data by white color (▭). (**A**) A panoramic dashboard was constructed to calculate the median value for each soluble mediator further organized in ascendant manner. (**B**) Scattering of individual values over bar charts underscore the decreased (shades of green), unaltered (gray) and increased (shades of red) median baseline fold change values. Dashed lines indicate the cut-offs employed to classify values according to distinct color shades (0.8, 1.0 and 1.5).

To further characterize the overall profile of systemic soluble mediators following 17DD-YF 1/5 fractional dose primary vaccination, the baseline fold change of each soluble mediator was determined for individual samples as the ratio of values observed for PV_(D30–45)_ divided by those detected for NV_(D0)_ referred as: PV_(D30–45)_/NV_(D0)_. The results are shown in Fig. 3. Panoramic heatmap dashboards of changes in systemic soluble mediators were assembled to calculate the median values of change for each soluble mediator further categorized as decreased, unaltered or increased values (Fig. 3A). The analysis of median values of change in soluble mediators indicated that amongst those molecules with decreased levels (median < 1.0), sFAS, CXCL5, TSLP, Trail and IL-18 presented a more prominent decrease (median < 0.8×) (Fig. 3B—dark green bars). Moreover, amongst those molecules with increased fold changes, IFN-γ, IL-3, TNF-α, CXCL8, CCL4, IL-6, CCL2, IL-1Ra, IL-1β e CCL7 exhibited a more prominent change (median > 1.5×). These findings pointed out that, within the mixed profile of systemic soluble mediators elicited by 17DD-YF 1/5 fractional dose primary vaccination, IFN-γ and TNF-α were included amongst the molecules with high baseline fold changes (Fig. 3B—dark red bars).

### Network mapping of systemic soluble mediators in adults following 17DD-YF 1/5 fractional dose primary vaccination

**Figure 4:**
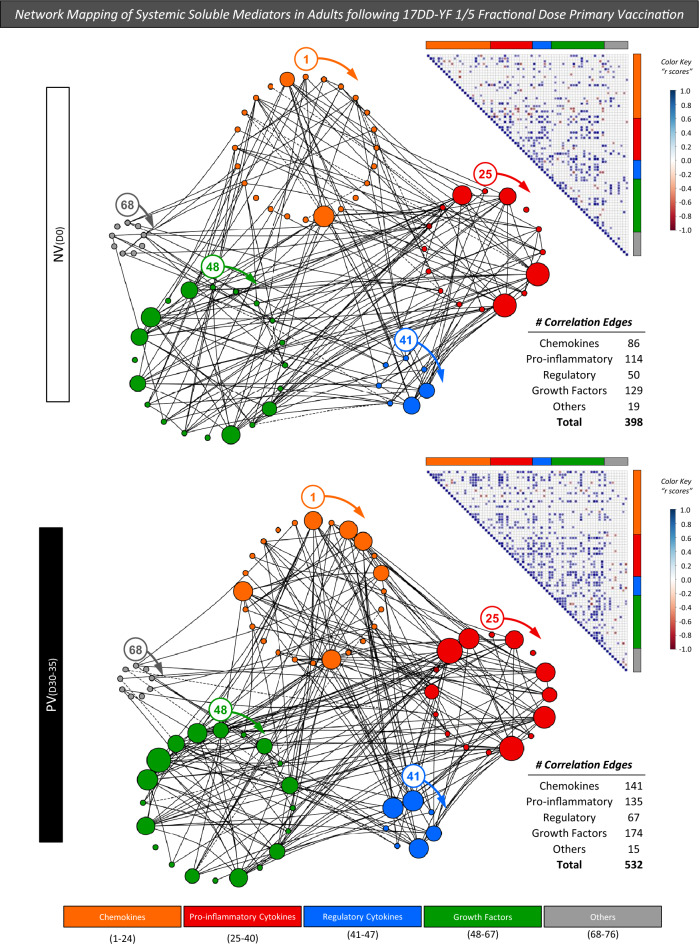
Network mapping of systemic soluble mediators in adults following 17DD-YF 1/5 fractional dose primary vaccination. Integrative networks were assembled for systemic soluble mediators observed in plasma samples obtained before [NV_(D0)_, n = 15] and 30–45 days after [PV_(D30–45)_, n = 15] 17DD-YF 1/5 fractional dose primary vaccination. The measurements were performed by xMAP technology as described in “Methods” section. Data analysis was carried out by Pearson and Spearman rank tests and only significant strong correlations (p < 0.05 and “r” scores ≥|0.67|) employed to assemble the integrative network mapping. Cluster layout networks were build, comprising 5 categories as follows: chemokines ( = 1–24; CCL1, CCL2, CCL3, CCL4, CCL5, CCL7, CCL8, CCL11, CCL13, CCL15, CCL17, CCL21, CCL22, CCL24, CCL26, CCL27, CXCL1, CXCL5, CXCL8, CXCL9, CXCL10, CX3CL1, CXCL12, CXCL13); pro-inflammatory cytokines ( = 25–39; IFN-⍺2, IL-28A, IL-1⍺, IL-1β, IL-6, IL-20, TNF-⍺, TNF-β, IL-12P40, IL-12P70, IFN-γ, IL-15, IL-17A, IL-18, IL-22, IL-23), regulatory cytokines ( = 40–47; IL-1Ra, IL-4, IL-5, IL-9, IL-10, IL-13, TGF-⍺), growth factors ( = 48–67; FGF-β, HGF, PDGF-AA, PDGF-AB/BB, VEGF-A, EGF, FLT-3L, SCF, IL-3, TPO, G-CSF, GM-CSF, IL-2, IL-7, IL-16, IL-21, IL-33, TSLP, MIF, LIF) and others ( = 68–76; Resistin, Leptin, sCD40L, sICAM1, sVCAM-1, sFAS, sFASL, Trail, PAI-1). Nodes with more than 10 strong correlations (p < 0.05 and “r” scores ≥|0.67|) were resized proportionally. Correlation matrices illustrate a panoramic overview of correlation between pairs of soluble mediators. The “r” scores, ranging from − 1 to + 1, are underscored according to the color key provided in the figure. The total number of strong correlations and those observed for each cluster were used for comparative analysis between NV_(D0)_ and PV_(D30–45)_.

The use of high-throughput technologies, such multiplex analysis of soluble immune mediators combined with computational modeling tools have been currently applied in vaccinology to identify immune response signatures. In this sense, correlation analysis between pairs of soluble mediators has been used to assemble correlogram matrices and build integrative network mappings. The correlation numbers between systemic soluble mediators were assessed for NV_(D0)_ and PV_(D30–45)_ and the results shown in Fig. 4. 17DD-YF 1/5 fractional dose primary vaccination induced an increase in the number of correlation edges in most systemic soluble mediators as observed in PV_(D30–45)_ as compared to NV_(D0)_ (532 *vs* 398 connections, respectively). Changes from NV_(D0)_ to PV_(D30–45)_ were characterized by a more prominent increase in the correlation numbers within the chemokine cluster (86 to 141; 1.6×) followed by growth factors (129 to 174; 1.4×), pro-inflammatory cytokines (108 to 135; 1.3×), regulatory cytokines (56 to 67; 1.3×) (Fig. 4). Overall, the integrative network mapping showed an increase in the correlation numbers between soluble mediators elicited by 17DD-YF 1/5 fractional dose primary vaccination.

### Phenotypic features of YF-specific cellular response in adults following 17DD-YF 1/5 fractional dose primary vaccination

**Figure 5:**
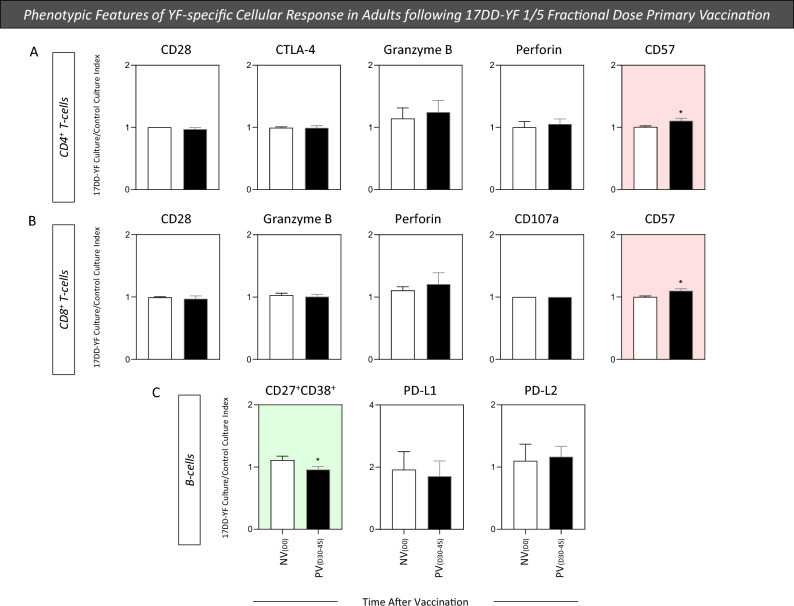
Phenotypic features of YF-specific cellular response in adults following 17DD-YF 1/5 fractional dose primary vaccination. The YF-specific phenotypic features of T and B-cells were evaluated upon long-term in vitro PBMC cultures carried out before [NV_(D0)_ = ▭, n = 15] and 30–45 days after [PV_(D30–45)_ = ▬, n = 15] 17DD-YF 1/5 fractional dose primary vaccination. PBMC cultures were performed in the absence (unstimulated—Control Culture) and in the presence of 17DD-YF antigen (17DD-YF stimulated—17DD-YF Culture). Immunophenotyping staining of cell-surface markers (CD28, CTLA-4, Granzyme-B, Perforin, CD57 for T-cells and CD27, CD38, PD-L1, PD-L2 for B-cells) was carried out by mass cytometry by time-of-flight (CyTOF) as described in “Methods” section. Results are shown as mean values ± standard error of 17DD-YF Culture/Control Culture Index. Comparative analysis of YF-specific phenotypic features of CD4^+^ (**A**), CD8^+^ T-cells (**B**) and B-cells (**C**) between NV_(D0)_ and PV_(D30–45)_ was carried out by Student t test. Significant differences were considered at p ≤ 0.05 and highlighted by *. Color background underscores cell phenotypes with increased (pink) or decreased (green) values in PV_(D30–45)_ as compared to NV_(D0)_.

The cell-mediated immune response has been considered a relevant biological marker to define proxies of immunoprotection induced by vaccines. Aiming at characterizing the YF-specific phenotypic features of T-cell subsets and B-cells, mass cytometry (CyTOF) immunophenotyping was carried out upon in vitro 17DD-YF antigen recall. The results, reported as 17DD-YF Culture/Control Culture Index for NV_(D0)_ and PV_(D30–45)_, are presented in Fig. 5. 17DD-YF 1/5 fractional dose induced an increase in CD57 expression by CD4^+^ and CD8^+^ T-cells and a decrease in CD27^+^CD38^+^ B-cells (plasmablasts) PV_(D30–45)_ as compared to NV_(D0)_ (Fig. 5). These results highlighted that both CD4^+^ and CD8^+^ T cells respond to 17DD-YF 1/5 fractional dose primary vaccination.

### YF-specific memory response in adults following 17DD-YF 1/5 fractional dose primary vaccination

**Figure 6:**
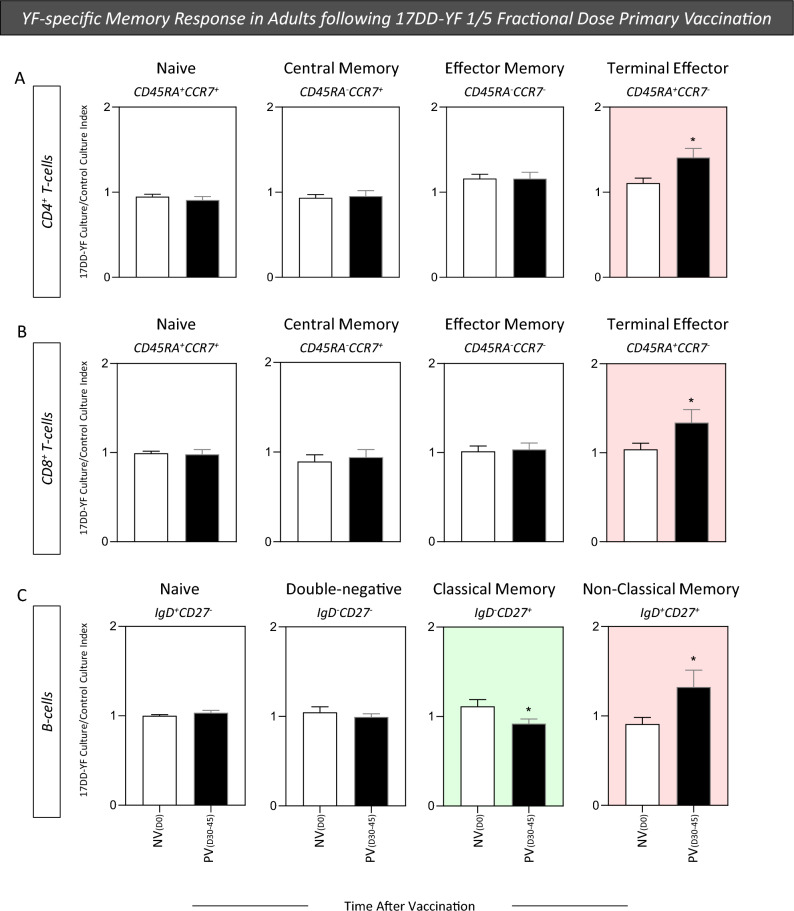
YF-specific memory response in adults following 17DD-YF 1/5 fractional dose primary vaccination. The YF-specific memory phenotypes of T and B-cells were evaluated upon long-term in vitro PBMC cultures carried out before [NV_(D0)_ = ▭, n = 15] and 30–45 days after [PV_(D30–45)_ = ▬, n = 15] 17DD-YF 1/5 fractional dose primary vaccination. PBMC cultures were performed in the absence (unstimulated—Control Culture) and in the presence of 17DD-YF antigen (17DD-YF stimulated—17DD-YF Culture). Immunophenotyping staining of memory phenotypes of CD4^+^ and CD8^+^ T-cell subsets (Naïve—CD45RA^+^CCCR7^+^; Central Memory—CD45RA^−^CCCR7^+^; Effector Memory—CD45RA^−^CCCR7^−^ and Terminal Effector—CD45RA^+^CCCR7^−^) and B-cells (Naive—IgD^+^CD27^−^; Double-negative—IgD^−^CD27^−^; Classical Memory—IgD^−^CD27^+^ and Non-Classical Memory—IgD^+^CD27^+^) was carried out by mass cytometry by time-of-flight (CyTOF) as described in “Methods” section. Results are shown as mean values ± standard error of 17DD-YF Culture/Control Culture Index. Comparative analysis of YF-specific memory phenotypes of CD4^+^ (**A**), CD8^+^ T-cells (**B**) and B-cells (**C**) between NV_(D0)_ and PV_(D30–45)_ was carried out by Student t test. Significant differences were considered as p ≤ 0.05 and highlighted by *. Color background underscores cell phenotypes with increased (pink) or decreased (green) values in PV_(D30–45)_ as compared to NV_(D0)_.

Previous studies have demonstrated that YF-vaccination can elicit distinct memory T and B-cell subsets, displaying conventional markers including central memory (TCM) and effector memory (TEM/TEMRA) as well as conventional and non-conventional memory subsets, respectively. In order to characterize the T and B-cell memory phenotypes, mass cytometry (CyTOF) immunophenotyping was carried out upon in vitro 17DD-YF antigen recall to define memory subsets of YF-specific cells, using CD45RA and CCR7 to quantify memory subsets within CD4^+^ and CD8^+^ T-cells and IgD and CD27 for B-cells. The results are reported as 17DD-YF Culture/Control Culture Index for NV_(D0)_ and PV_(D30–45)_ in Fig. 6. Data analysis demonstrated that 17DD-YF 1/5 fractional dose primary vaccination induced an increase of Terminal Effector (CD45RA^+^CCR7^−^) CD4^+^ and CD8^+^ T-cells along with a decrease of Classical (IgD^−^CD27^+^) and an increase of Non-Classical memory B-cells (IgD^+^CD27^+^) (Fig. 6).

**Figure 7:**
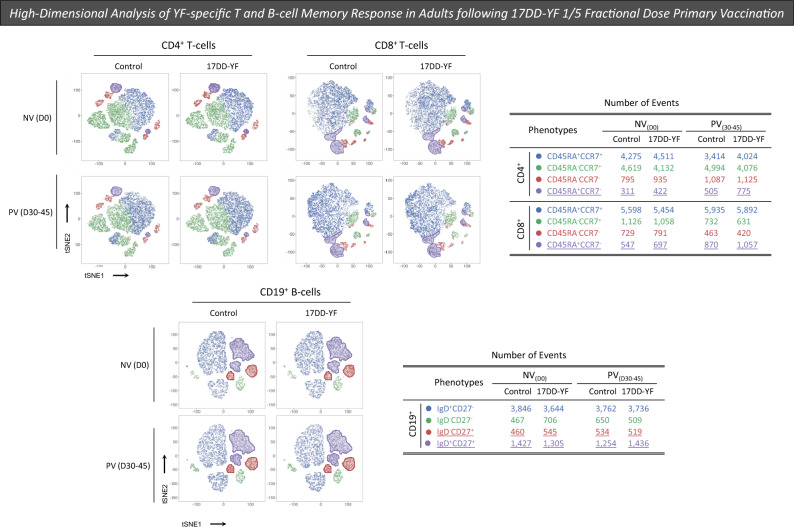
High-dimensional analysis of YF-specific T-cell memory response in adults following 17DD-YF 1/5 fractional dose primary vaccination. T-cell memory profile was characterized upon long-term in vitro PBMC cultures carried out before [NV_(D0)_ = ▭, n = 15] and 30–45 days after [PV_(D30–45)_ = ▬, n = 15] 17DD-YF 1/5 fractional dose primary vaccination. PBMC cultures were performed in the absence (unstimulated—Control Culture) and in the presence of 17DD-YF antigen (17DD-YF stimulated—17DD-YF Culture). Immunophenotyping staining of memory phenotypes of CD4^+^ and CD8^+^ T-cell subsets (Naïve—CD45RA^+^CCCR7^+^ = ; Central Memory—CD45RA^−^CCCR7^+^ = ; Effector Memory—CD45RA^−^CCCR7^−^ =  and Terminal Effector—CD45RA^+^CCCR7^−^ = ) as well as B-cells (Naive—IgD^+^CD27^−^ = ; Double-Negative—IgD^−^CD27^−^ = ; Classical Memory—IgD^−^CD27^+^ =  and Non-Classical Memory—IgD^+^CD27^+^ = ) was carried out by mass cytometry by time-of-flight (CyTOF) as described in “Methods” section. Dimensionality reduction of Mass Cytometry data was performed using tSNE (t-distributed Stochastic Neighbor Embedding) to visualize high-dimensional data of memory T and B-cells with supervised selection strategy as described in “Methods” section. A total of 5000 iterations were carried out using CD3, CD4 or CD8, CD45RA and CCR7 parameters for T-cells and CD19, CD20, CD21, IgD and CD27 parameters for B-cells. The hyperparameters tSNE_1 and tSNE_2 were visualized as heatmaps. The number of events for each memory cell phenotype was calculated considering a total of 10,000 events for CD4^+^ T-cells, 8000 for CD8^+^ T-cells and 6200 for B-cells.

Complementary analysis of the phenotypic profile of YF-specific cellular response was performed using tSNE (t-distributed Stochastic Neighbor Embedding) to visualize high-dimensional data of memory T and B-cells. Dimensionality reduction of Mass Cytometry data, using a supervised selection strategy, further illustrate that 17DD-YF 1/5 fractional dose primary vaccination induce increased levels of TEMRA CD4^+^ and CD8^+^ T-cells along with an increase of Non-Classical memory B-cells (Fig. 7).

### Functional profile of YF-specific cellular response in adults following 17DD-YF 1/5 fractional dose primary vaccination

**Figure 8:**
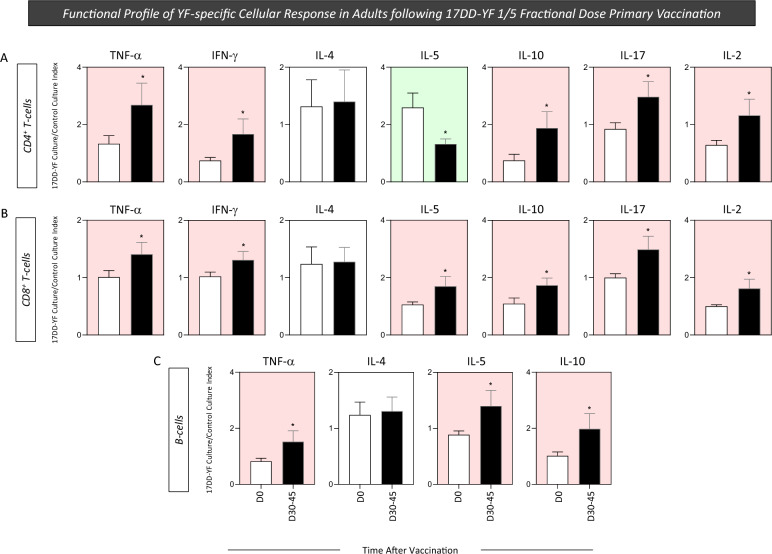
Functional profile of YF-specific cellular response in adults following 17DD-YF 1/5 fractional dose primary vaccination. The YF-specific functional profile of T and B-cells were evaluated upon long-term in vitro PBMC cultures carried out before [NV_(D0)_ = ▭, n = 15] and 30–45 days after [PV_(D30–45)_ = ▬, n = 15] 17DD-YF 1/5 fractional dose primary vaccination. PBMC cultures were performed in the absence (unstimulated—Control Culture) and in the presence of 17DD-YF antigen (17DD-YF stimulated—17DD-YF Culture). Intracytoplasmic cytokine staining (TNF-α, IFN-γ, IL-4, IL-5, IL-10, IL-17 and IL-2 for T-cells and TNF-α, IL-4, IL-5 and IL-10 for B-cells) was carried out by mass cytometry by time-of-flight (CyTOF) as described in “Methods” section. Results are shown as mean values ± standard error of 17DD-YF Culture/Control Culture Index. Comparative analysis of functional profile of CD4^+^ (**A**), CD8^+^ T-cells (**B**) and B-cells (**C**) between NV_(D0)_ and PV_(D30–45)_ was carried out by Student t test. Significant differences were considered at p ≤ 0.05 and highlighted by *. Color background underscores cell phenotypes with increased (pink) or decreased (green) values in PV_(D30–45)_ as compared to NV_(D0)_.

The analysis of cytokine produced by distinct cell subsets is relevant to provide additional information to understand in more detail the role of adaptive immune response elicited by vaccination. In this study, additional analysis of YF-specific cellular response was performed upon in vitro 17DD-YF antigen recall, focusing on pro-inflammatory and regulatory cytokines, to characterize the functional features of T-cell subsets and B-cells. The results are reported as 17DD-YF Culture/Control Culture Index for NV_(D0)_ and PV_(D30–45)_ in Fig. 8. 17DD-YF 1/5 fractional dose primary vaccination induced an increase of TNF-α, IFN-γ, IL-10, IL-17, IL-2 and decreased levels of IL-5 in CD4^+^ T-cells (Fig. 8A). Moreover, an overall increase of cytokine^+^ cells was observed for CD8^+^ T-cells and B-cells (Fig. 8B,C, respectively). No changes were observed for IL-4 production in T-cell subsets and B-cells (Fig. 8). Our findings demonstrated that 17DD-YF 1/5 fractional dose primary vaccination induced a polyfunctional cytokine profile of T-cell subsets and B-cells, mediated by increased levels of IFN-γ and TNF-α and lower production of IL-10.

### In vitro timeline kinetics of antigen-specific production of soluble mediators by PBMC from adults following 17DD-YF 1/5 fractional dose primary vaccination

**Figure 9:**
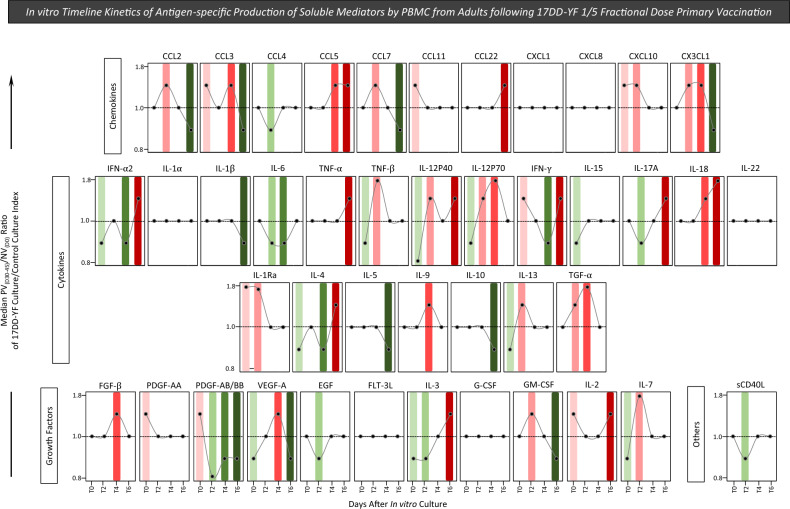
In vitro timeline kinetics of antigen-specific production of soluble mediators by PBMC from adults following 17DD-YF 1/5 fractional dose primary vaccination. Soluble mediators were measured in supernatants from in vitro PBMC cultures carried out before [NV_(D0)_, n = 15] and 30–45 days after [PV_(D30–45)_, n = 15] 17DD-YF 1/5 fractional dose primary vaccination. PBMC cultures were performed in the absence (unstimulated—Control Culture) and in the presence of 17DD-YF antigen (17DD-YF stimulated—17DD-YF Culture) and supernatants collected along the timeline kinetics (T0, T2, T4 and T6) for soluble mediators measurements by Luminex xMAP technology, as described in “Methods” section. The results are presented in line charts as median values of PV_(D30–45)_/NV_(D0)_ ratio along the timeline kinetics. Color rectangles were used to underscore the soluble mediators with decreased (median < 1; ) or increased (median > 1; ) values.

**Figure 10:**
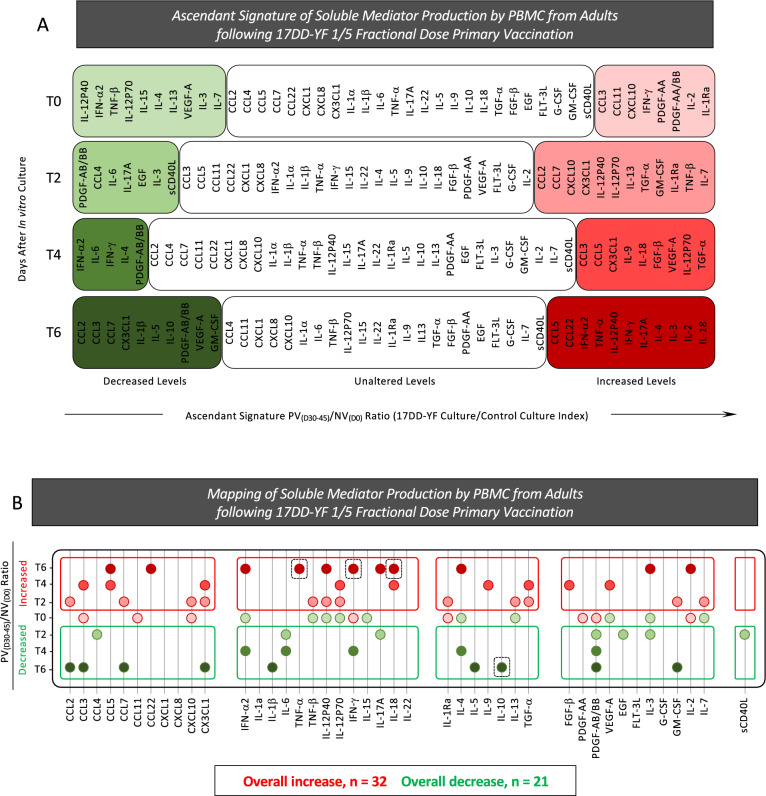
Ascendant signature and mapping of soluble mediator production by PBMC from adults following 17DD-YF 1/5 fractional dose primary vaccination. Soluble mediators were measured in supernatants from in vitro PBMC cultures carried out before [NV_(D0)_, n = 15] and 30–45 days after [PV_(D30–45)_, n = 15] 17DD-YF 1/5 fractional dose primary vaccination. PBMC cultures were performed in the absence (unstimulated—Control Culture) and in the presence of 17DD-YF antigen (17DD-YF stimulated—17DD-YF Culture) and supernatants collected along the timeline kinetics (T0, T2, T4 and T6) for soluble mediators measurements by Luminex xMAP technology, as described in “Methods” section. (**A**) Ascendant signature of soluble mediator production by PBMC were assembled to identify the set of biomarkers with decreased (green background; ), unaltered (clear background; ▭) or increased (red background; ) values along the timeline kinetics. (**B**) Mapping of soluble mediator production by PBMC were drawn to compile the soluble mediators with selective increase (red circles; ) or decrease (green circles; ) values along the timeline kinetics. The total number of soluble mediators with overall increase or decrease are provided in the figure. The increased levels of TNF-α, IFN-γ and decreased levels of IL-10 observed at T6 are highlighted by dashed frame.

Studying the kinetics of soluble mediator production in cell culture supernatants upon in vitro 17DD-YF antigen recall is a rational strategy to characterize the cell-to-cell communication underlying the assembling of antigen-specific immune responses. In order to characterize the dynamic of soluble mediators production upon in vitro 17DD-YF antigen recall, cell culture supernatants were analyzed by high-throughput microbeads xMAP technology to measure 43 analytes along the timeline kinetics (T0, T2, T4 and T6). The results are reported as median values of PV_(D30–45)_/NV_(D0)_ ratio along the timeline kinetics as presented in Fig. 9. In vitro 17DD-YF antigen recall induced a complex and polyfunctional profile of soluble mediator, characterized by increased PV_(D30–45)_/NV_(D0)_ ratio from T2 towards T6 (Fig. 9, red rectangles). Aiming to further characterize the overall profile of soluble mediator production, data were assembled as ascendant signature to identify sets of soluble mediators with decreased (median < 1; green backgrounds), unaltered (median = 1; clear backgrounds) or increased (median > 1; red backgrounds) values along the timeline kinetics (Fig. 10A). The map of soluble mediator production was drawn to compile those molecules with selective increased or decreased values from T2 towards T6 as reported in the Fig. 10B. Data demonstrated a predominance of increased (n = 32) over decreased (n = 21) PV_(D30–45)_/NV_(D0)_ ratio along the timeline kinetics (Fig. 10B). In details, soluble mediators with increased levels comprises: CCL2, CCL7, CXCL10, CX3CL1, TNF-β, IL-12P40, IL-12P70, IL-1Ra, IL-13, TGF-α, GM-CSF and IL-7 at T2; CCL3, CCL5, CX3CL1, IL-12P70, IL-18, IL-9, TGF-α, FGF-β and VEGF-A at T4 and CCL5, CCL22, IFN-α2, TNF-α, IL-12P40, IFN-γ, IL-17A, IL-18, IL-4, IL-3 and IL-2 at T6. Soluble mediators with decreased levels included: CCL4, IL-6, IL-17A, PDGF-AB/BB, EGF, IL-3 and sCD40L at T2; IFN-α2, IL-6, IFN-γ, IL-4 and PDGF-AB/BB at T4 and CCL2, CCL3, CCL7, CX3CL1, IL-1β, IL-5, IL-10, PDGF-AB/BB and GM-CSF at T6 (Fig. 10B). Of note, was the increased levels of TNF-α and IFN-γ with a decreased levels of IL-10 observed at T6 (Fig. 10B, dashed frame). Together, these findings demonstrated a complex and polyfunctional profile of soluble mediator is required to assemble the antigen-specific immune response elicited by 17DD-YF 1/5 fractional dose primary vaccination.

## Discussion

This is a pioneer investigation that provided a comprehensive landscape of the immune response features elicited by the 1/5 fractional dose of 17DD-YF primary vaccination in adults during a large-scale vaccination campaign in Brazil, employing powerful and advanced high-dimensional tools for immune response characterization.

Despite the availability of YF vaccine since 1937, which has consistently demonstrated safety and efficacy outcomes, outbreaks have increasingly been reported in tropical areas in the last decades. Notably, the disease has emerged in regions with historically low circulation of YF virus, underscoring YF as a globally significant infectious disease with public health implications^5,6,13^. In these areas, the low vaccination coverage facilitates the introduction of the YF virus, resulting in a substantial population lacking protection against the virus.

As a result of the YF epidemiological scenario, there was a sharp increase in demand for YF vaccine, leading to a global shortage stockpile. In an effort to enhance vaccine availability and prevent or control YF outbreaks, the WHO conducted a comprehensive evaluation of 17DD-YF fractional dose efficacy. This approach has been used as a criterion to recommend the large-scale campaigns in response to emergency situations^14,15^.

In order to bring novel insights regarding the use of fractional dose of 17DD-YF vaccine, the present study aimed at evaluating the neutralizing antibody profile as well as a multiparametric analysis of cell-mediated response elicited by the 1/5 fractional dose of 17DD-YF primary vaccination in adults.

A few studies have evaluated the immune response triggered by subdoses (decreasing number of 17DD-YF viral particles)^16,17^ or fractional dose^11^ of the 17DD-YF vaccine, indicating consistent seroconversion rates when compared to standard dose. In our study, primary vaccination with 1/5 fractional dose of 17DD-YF vaccine was found to induce a significant increase of YF-specific neutralizing antibody titers (GMT = 690.7) with outstanding seropositivity rate (93%) at PV_(D30–45)_ compared to NV_(D0)_. Altogether, these results demonstrated that 1/5 fractional dose of the 17DD-YF vaccine induces significant seroprotection in primary adult vaccinees, as supported by previous studies^11,17^. In addition, it has been strongly established that neutralizing antibodies is an important correlate of protection for YF vaccination^18,19^.

Besides the importance of neutralizing antibodies to guarantee vaccine immune protection^20–22^, analysis of cell-mediated features is also relevant to define immunoprotection followed by 17DD-YF vaccine^16^. Some studies have confirmed the importance of cellular immunity components in the 17DD-YF vaccination context, indicating the necessity of a mixed microenvironment with activation/modulation features as well as a balanced cytokine/chemokine response throughout the vaccination kinetics^16,23,24^.

In this context, we assessed the changes of 76 systemic soluble mediators following 17DD-YF 1/5 fractional dose primary vaccination. Our results demonstrated that the primary vaccination displayed a mixed profile of systemic soluble mediators at PV_(D30–45)_ when compared to NV_(D0)_, with IFN-γ and TNF-α included within the molecules with high baseline fold changes. Likewise, it has been demonstrated that a dynamic profile, including regulatory and pro-inflammatory cytokines, is essential to generate a robust immune response elicited by standard dose of the 17DD-YF vaccine^16,25^. In this matter, it has been described that the 17DD-YF vaccine induces a pro-inflammatory profile with intensified production of IFN-γ and TNF-α. These molecules are important in aiding neutralizing antibody production, conferring protection against the YF virus and guaranteeing efficacy after vaccination^25^.

Previous studies that evaluated the standard dose of 17DD-YF primary vaccination in adults have also demonstrated important elements of T-cell subsets and B-cell memory-related phenotypes, including increased levels of Effector Memory (CD27^−^CD45RO^+^) CD8^+^ T-cells and Non-Classical memory B-cells (IgD^+^CD27^+^) 30–45 days after vaccination^16,26–28^. In the present study we have assessed additional memory phenotypic profiles, and showed that increased levels of Terminal Effector (CD45RA^+^CCR7^−^) of CD4^+^ and CD8^+^ T-cells and Non-Classical memory B-cells (IgD^+^CD27^+^) were observed in PV_(D30–45)_, supporting that 1/5 fractional dose of 17DD-YF primary vaccination can effectively induce memory phenotypes likewise observed for standard dose.

Complementary analysis of the functional profile of YF-specific cellular response was also evaluated in this study after 17DD-YF 1/5 fractional dose primary vaccination in adults. In agreement with a previous study that evaluated the immune response of the standard dose of 17DD-YF vaccine, our results demonstrated a polyfunctional cytokine profile of T-cell subsets and B-cells upon in vitro 17DD-YF antigen recall, mediated by increased levels of IFN-γ and TNF-α along with decreased production of IL-10^26,29^, confirming that 1/5 fractional dose of 17DD-YF primary vaccination exhibited a similar immune response compared to standard dose.

It is important to highlight that this study has some limitations. The non-probabilistic sampling of participants with distinct unverified characteristics that might affect their immune responses may impact the results. However, there were explicit recommendations to exclude individuals with acute infections or chronic clinical conditions, such as those that include the use of immunosuppressants. The small sample size selected for the detailed cell-mediated immunological studies represented another limitation of the study. The lack of previous studies focusing on the cellular immune response elicited by fractional dose can also be pointed out as a limitation for comparative analysis. Moreover, in the present study, the comparative analysis of the immune response induced by 1/5 fractional and the standard dose was not performed, since during the vaccination campaign, the Brazilian Health Ministry has determined that only fractional dose was available for the general population resident in the risk areas. Therefore, in order to overcome this limitation, we have interpreted the current results in the light of previous studies carried out with the standard dose^11,16–19,23–28^. It is important to mention that in the present study, the 1/5 fractional dose of 17DD-YF vaccine was administered as a single subcutaneous injection. A previous study has indicated that YF-specific neutralizing antibody titers did not differ between volunteers receiving intradermal 1/5 fractional dose and subcutaneous standard dose^32^. However, considering that distinct administration routes of live-attenuated YF-vaccine may impact the cell-mediated immune response, further studies addressing this issue are still required.

Among the implications of our results, the role of memory cells and a crucial role of pro-inflammatory cytokines in producing and maintaining the effectiveness of neutralizing antibody activity can be highlighted.

In conclusion, the present study demonstrated that 1/5 fractional dose of 17DD-YF vaccine was able to induce a robust immune response in adults. The μFRN-HRP levels and seropositivity rates along with the cellular immune response provide relevant evidence to support the use of 1/5 fractional dose of 17DD-YF vaccine in adults in emergency situations. Further studies are needed to monitor the duration of YF-specific memory immunity to verify whether or when primary vaccinees who received 17DD-YF 1/5 fractional dose need to be revaccinated to guarantee long-term immunity to yellow fever virus. Moreover, studies to evaluate the fractional-dose vaccination response in children and special groups including pregnant women and persons living with the human immunodeficiency virus are still required.

## Methods

### Study population and design

**Figure 11:**
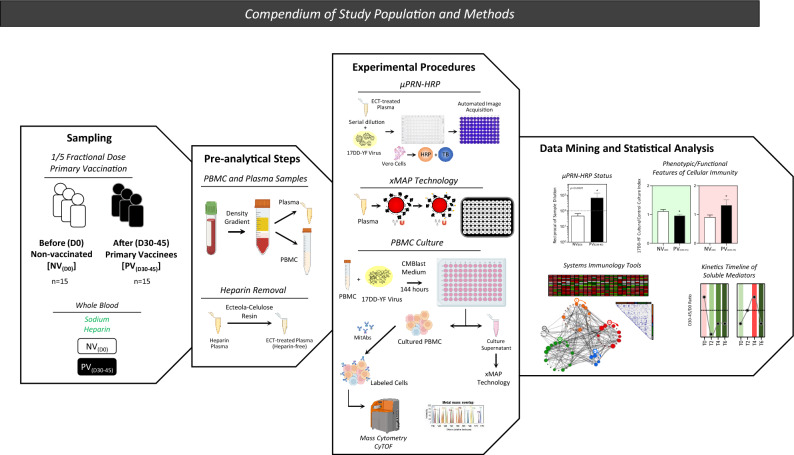
Compendium of study population and methods. An overview of sampling, pre-analytical steps, experimental procedures, data mining and statistical analysis employed in the present investigation. The study population was composed of 15 subjects, both sexes, age ranging from 18 to 57 years old. Whole blood samples were collected from each volunteer before [NV_(D0)_] and 30–45 days after [PV_(D30–45)_] 17DD-YF 1/5 fractional dose primary vaccination in vacuum system containing sodium heparin as anticoagulant. PBMC and plasma samples were isolated by Histopaque-1077 density gradient. Plasma samples were pre-treated with ecteola-cellulose for heparin removal. Heparin-free plasma were used for detection of YF-specific neutralizing antibodies by micro Focus Reduction Neutralization—Horseradish Peroxidase (μFRN-HRP) test. Plasma aliquot was employed to characterize the profile of systemic soluble mediators by Luminex xMAP technology. PBMC samples were submitted to long-term in vitro culture to evaluate YF-specific phenotypic/functional features of cellular immunity by Mass Cytometry (CyTOF) using a cocktail of metal-isotope-tagged antibodies (MitAbs). Cell culture supernatant was collected to quantify soluble mediators by high-throughput microbeads xMAP technology. Distinct approaches were used for data mining and statistical analysis, including: quantification of neutralizing antibodies (µFRN-HRP status), analysis of phenotypic/functional features of cellular immunity, use of systems immunology tools and kinetics timeline of soluble mediators.

This study is an observational investigation carried out by the Collaborative Group for Studies of Yellow Fever Vaccine between January to July 2018 in Rio de Janeiro, Brazil, during a large-scale vaccination campaign with 1/5 fractional dose of 17DD-YF vaccine. The 1/5 fractional dose of 17DD-YF vaccine was administered as a single subcutaneous injection of 0.1 mL of reconstituted vaccine. The study population enrolled a convenience sample from the major study comprised of 15 individuals, from both genders, with ages ranging from 18 to 57 years. Biological samples were obtained from each volunteer at two consecutive time points: before (D0) and 30–45 days after (D30–45) 17DD-YF 1/5 fractional dose primary vaccination, referred as: non-vaccinated [NV_(D0)_] and primary vaccinees [PV_(D30–45)_], respectively. Figure 11 summarizes the study population and methods.

The study protocol was submitted and approved by the ethical committee at Instituto René Rachou—Fundação Oswaldo Cruz (CAAE: 82357718.5.0000.5091), Secretaria Municipal da Saúde de São Paulo—SMS/SP (CAAE: 82357718.5.3003.0086), Instituto Nacional de Infectologia Evandro Chagas—INI/FIOCRUZ (CAAE: 82357718.5.3001.5262) and Instituto de Infectologia Emílio Ribas—IIER (CAAE: 82357718.5.3002.0061). All participants have read and signed the written/informed consent prior to inclusion in the study. This investigation fulfills the principles of the Helsinki declaration and the 466/2012 resolution from the Brazilian National Health Council for research involving human subjects.

### Biological samples for mononuclear cells and plasma isolation

Peripheral blood samples (10–20 mL) were collected at distinct time points (D0 and D30-45) from all participants by venipuncture using a vacuum system containing spray-dried sodium heparin as anticoagulant. Blood specimens were employed to obtain plasma samples and peripheral blood mononuclear cells (PBMC). Plasma specimens were collected from the top layer of whole blood after centrifugation at 3000 rpm for 10 min, at room temperature, aliquoted and stored at − 80 °C for further analysis of YF-specific neutralizing antibodies and systemic soluble mediators. After plasma removal, samples were reconstituted to original volume in RPMI-1640, followed by PBMC isolation using Histopaque-1077 density gradient upon centrifugation at 1800 rpm for 40 min, at room temperature. PBMC aliquots (1 × 10^7^ cells/mL) were stored at − 80 °C for further analysis of YF-specific phenotypic/functional features of cellular immunity.

### Analysis of YF-specific neutralizing antibodies

Plasma samples stored at − 80 °C were thawed at 37 °C and pre-treated with ecteola-cellulose for heparin removal as previously described by Campi-Azevedo et al.^30^, prior use for the YF-specific micro Focus Reduction Neutralization—Horseradish Peroxidase (μFRN-HRP). The μFRN-HRP test was carried out at Laboratório de Análise Imunomecular, Bio-Manguinhos (LANIM, FIOCRUZ-RJ, Brazil) to quantify the titers of YF-specific neutralizing antibodies and define the seropositivity rates according to Simões^12^, as follows: briefly, in 96-well plates, a mixture of ECT-treated plasma samples (1:6 to 1:1458) plus 17D-213/77 vaccine virus (70 PFU/well) was pre-incubated (2 h; 37 °C; 5% CO_2_) and transferred to pre-formed Vero ATCC cell monolayer. Carboxymethylcellulose semisolid medium (2%) were added to each well and monolayers incubated for 48 h at 37 °C, 5% CO_2_. Thereafter, cells were washed, fixed with ethanol/methanol (1:1) solution and incubated with 4G2 HRP-conjugated monoclonal antibody (2 h; 35 °C; 5% CO_2_) and True Blue Dye substrate. After incubation, monolayers were washed, dried and photographed using the ScanLab microscope with automated counting. The neutralizing antibody titers were determined as the last ECT-treated plasma dilution able to reduce the number of plaques by 50% (EP_50_) as compared to control. Seropositivity rate was calculated considering the antibody titer ≥ 100 as the cut-off.

### Long-term in vitro cultures of peripheral blood mononuclear cells

PBMC samples stored at − 80 °C were processed according to Lin et al.^31^, modified as follows: PBMC samples were thawed in water bath at 37 °C and resuspended in 11 mL warm (37 °C) supplemented RPMI-1640 (5% heat-inactivated AB normal human serum; 10,000 IU/mL penicillin; 10 mg/mL streptomycin; 0.025 mg/mL amphotericin B and 2 mM l-glutamine) containing 25 U/mL benzonase. Cells were washed (2000 rpm; 10 min; room temperature) and resuspended in supplemented RPMI-1640, counted in the Vi-CELL™ Cell Viability Analyzer counter and adjusted to a final concentration of 2 × 10^7^ cells/mL. Cells were kept on ice until the start of long-term in vitro cultures.

Long-term PBMC cultures were carried out as previously described by Costa-Pereira et al.^26^, as follows: PBMC aliquots (1.0 × 10^6^ cells/well) were incubated in the presence of 200 μL of supplemented RPMI-1640. Parallel batches of unstimulated (Control Culture—10 μL/well of RPMI-1640) and 17DD-YF antigen-stimulated (17DD-YF Culture—lyophilized 17DD-YF vaccinal live-attenuated virus reconstituted in RPMI-1640 to reach a final concentration of 250 LD_50_/10 μL/well) cultures were maintained for 6 days at 37 °C, 5% CO_2_. Aliquots of culture supernatants (100 μL) were collected at day 0 (T0), day 2 (T2), day 4 (T4) and day 6 (T6) for soluble mediator measurements and replaced with fresh supplemented RPMI-1640. At day 6, cultures were treated with Brefeldin-A (0.01 mg/mL) for 4 h and then incubated with 2 mM ethylenediamine-tetra-acetic acid for 15 min at room temperature. Following, cells were harvested and washed twice with CyFACS Buffer (PBS without heavy metal contaminants; 0.1% BSA; 2 mM EDTA; 0.05% sodium azide) for further immunostaining for phenotypic/functional features.

### Phenotypic and functional analysis by mass cytometry by time-of-flight (CyTOF)

Immunophenotyping staining was carried out as previously described by Lin et al.^31^ as follows: long-term in vitro cultured PBMC were incubated for 45 min on ice with a cocktail of metal-isotope-tagged antibodies (MitAbs), including: anti-CD3/UCHT1/154Sm, anti-CD4/RPA-T4/145Nd, anti-CD8/RPA-T8/146Nd, anti-CD45RA/HI100/153Eu, anti-CCR7/150503/169Tm; anti-CD19/HIB19/142Nd, anti-IgD/IA6-2/170Er, anti-CD20/2H7/147Sm; anti-CD21/Bu32/174Yb; anti-CD27/L128/167Er; anti-CD28/L283/155Gd, anti-CD57/HCD57/113In, anti-CD38/HB-7/156Gd, anti-PD-L1/29E.2A3/175Lu, anti-PDL-2/24F.10C12/172Yb, anti-CD14/M5E2/160Gd and CD33/WM53/158Gd. Stained cells were washed twice, incubated with live-dead Maleimide-DOTA/115In solution (1.6 μg/mL) and fixed with 2% paraformaldehyde overnight. Following, fixed PBMC were washed twice with permeabilization buffer and incubated with monoclonal antibodies cocktail (anti-GranzimaB/GB11/171Yb; anti-Perforin/B-D48/173Yb; anti-TNF-α/Mab11/152Sm; anti-IFN-α/4S.B3/161Dy; anti-IL-4/MP4-25D2/144Nd; anti-IL-5/BVD2-21C11/159Tb; anti-IL-10/JES3-9D7/143Nd; anti-IL-17/N49-853/164Dy; anti-IL-2/MQ1-17h12/166Er). Cells were washed, incubated with Ir-DNA Intercalator (191Ir/193Ir cocktail) and resuspended in Milli-Q water for CyTOF analysis. EQ Four Element Calibration Beads from Fluidigm (140Ce, 151Eu, 165Ho, 175Lu) were added as per the manufacturer’s directions prior to running. Data were acquired on a Helios mass cytometer (Fluidigm, San Francisco, CA, USA). Distinct gating strategies were used for phenotypic/functional analysis as illustrated by representative Cytobank plots in Supplementary Fig. 1.

### Analysis of soluble mediators in plasma and cell culture supernatant by xMAP Technology

The quantification of soluble mediators was carried out by high-throughput microbeads xMAP technology at Human Immune Monitoring Center (HIMC), Stanford University, CA, USA. Kits were purchased from EMD Millipore Corporation, Burlington, MA., and used according to the manufacturer’s recommendations with modifications described as follows: H76 kits included 3 panels: Panel 1 was Milliplex HCYTMAG60PMX41BK with IL-18 and IL-22 added to generate a 43 plex; Panel 2 was Milliplex HCP2MAG62KPX23BK with MIG/CXCL9 added to generate a 24 plex; and Panel 3 included the Milliplex HSP1MAG-63K with Resistin, Leptin and HGF add to generate a 9 plex. All three panels were used for analysis, only panel 1 (43-plex) was used for cell culture supernatant analysis. Assay setup was as follows: samples were mixed with antibody-linked magnetic beads on a 96-well plate and incubated overnight at 4 °C with shaking. Cold and room temperature incubation steps were performed on an orbital shaker at 500–600 rpm. Plates were washed twice with wash buffer in a Biotek ELx405 washer. Following one hour incubation at room temperature with biotinylated detection antibody, streptavidin-PE was added for 30 min with shaking. Plates were washed as above, and PBS added to wells for reading in the Luminex FlexMap3D Instrument with a lower bound of 50 beads per sample per cytokine. Each sample was measured in duplicate. Custom Assay Chex control beads were purchased from Radix Biosolutions, Georgetown, Texas, and are added to all wells. Quantitative data were reported as mean fluorescence intensity (MFI) or final concentration (pg/mL) estimated by 5-parameter logistic regression according to standard curves included in each experimental batch.

### Data analysis

Multiple strategies were applied for data mining and statistics. Comparative analysis of neutralizing antibody titers (Geometric Mean Titers, GMT) and seropositivity rates (percentage, %) observed at NV_(D0)_ and PV_(D30–45)_ were carried out by Student t-test and Chi-square test, respectively. Analysis of systemic soluble mediators between NV_(D0)_ and PV_(D30–45)_ was performed by Mann–Whitney test. In all cases, significant differences were considered when p ≤ 0.05.

The baseline fold change of systemic soluble mediators was determined for individual samples as the ratio of values observed for PV_(D30–45)_ divided by those detected for NV_(D0)_. The baseline fold changes values were used to construct a panoramic heatmap dashboard used to calculate the median values of change for each soluble mediator, further assembled in bar charts to underscore the molecules with decreased (median < 1.0), unaltered (median = 1.0) or increased (median > 1.0) values.

Correlation analysis (Pearson and Spearman rank test) was employed to assemble integrative correlation matrices to build integrative network mappings, considering only strong significant correlations (p < 0.05 and “r” scores ≥|0.67|) between pairs of attributes. The open-source Cytoscape platform (available at http://cytoscape.org) was used to generate cluster layout networks comprising 5 categories of systemic soluble mediators—chemokines, pro-inflammatory cytokines, regulatory cytokines, growth factors and others, with nodes representing each soluble mediator.

The analysis of YF-specific phenotypic features and memory profile of T and B-cells was performed by first converting the original continuous variable results into categorical data, expressed as 17DD-YF Culture/Control Culture Index. Comparative analysis between indices observed at NV_(D0)_ and PV_(D30–45)_ were carried out by Student t-test and significance considered at p ≤ 0.05.

Dimensionality reduction of Mass Cytometry data was performed using tSNE (t-distributed Stochastic Neighbor Embedding) to visualize high-dimensional data of memory T and B-cells. The analysis of memory subsets within CD4^+^ and CD8^+^ T-cells as well as B-cells was carried out using a supervised selection strategy. To reduce dimensionality, the fcs files were loaded into the R software using the Flowcore package. The expression intensity values were transformed by the hyperbolic arcsine function (arcsinh) and exported as csv files. Graphical representations were constructed based on algorithms implemented by the Rtsne package adjusted with perplexity values of 30 for CD4^+^ and B-cells and 50 for CD8^+^ T-cells. A total of 5000 iterations were carried out using CD3, CD4 or CD8, CD45RA and CCR7 parameters for T-cells and CD19, CD20, CD21, IgD and CD27 parameters for B-cells. The hyperparameters were visualized as tSNE_1 and tSNE_2 using the ggplot2 package.

The analysis of YF-specific functional profile of T and B-cells was performed by intracytoplasmic Mass Cytometry staining after long-term culture and by quantification of soluble mediators in cell culture supernatant along the timeline kinetics. In both cases, data analysis was performed by first converting the original continuous variable results into categorical data, expressed as 17DD-YF Culture/Control Culture Index (% of cells in 17DD-YF Culture/% of cells in Control Culture, calculated for D0 and D30-45). Comparative analysis of intracytoplasmic Mass Cytometry data observed at NV_(D0)_ and PV_(D30–45)_ were carried out by Student t-test and significance considered at p ≤ 0.05. The analysis of soluble mediators along the timeline kinetics was performed as biomarker signatures as follows: the 17DD-YF Culture/Control Culture Indices were used to further calculate the PV_(D30–45)_/NV_(D0)_ ratio values determined for individual samples. The median value of PV_(D30–45)_/NV_(D0)_ ratio for each soluble mediator obtained at baseline (T0), 48 h (T2), 96 h (T4) and 144 h (T6) were used to assemble the in vitro timeline kinetics. Comparative analysis (T0 *vs* T2 *vs* T4 *vs* T6) was carried out using the ascendant signature of soluble mediator production to identify molecules with decreased (median < 1), unaltered (median = 1) or increased (median > 1) values along the timeline kinetics. Mapping of soluble mediator production was employed to compile the soluble mediators with selective increase or decrease values from T2 towards T6.

### Supplementary Information

Supplementary Figures.

## Data Availability

The results included in the present study are available from the corresponding author [HTM and OAMF] upon request.

## References

[1] Cavalcante K, Tauil PL (2017). Risk of re-emergence of urban yellow fever in Brazil. Epidemiol. Serv. Saude.

[2] Douam F, Ploss A (2018). Yellow fever virus: Knowledge gaps impeding the fight against an old foe. Trends Microbiol..

[3] Monath TP, Vasconcelos PF (2015). Yellow fever. J. Clin. Virol..

[4] Campi-Azevedo AC (2016). Booster dose after 10 years is recommended following 17DD-YF primary vaccination. Hum. Vaccines Immunotherap..

[5] Maguire HC, Heymann DL (2016). Yellow fever in Africa. BMJ.

[6] Brandão-de-Resende C (2019). Characterization of retinopathy among patients with yellow fever during 2 outbreaks in Southeastern Brazil. JAMA Ophthalmol..

[7] PAHO/WHO. *Yellow Fever*. https://www3.paho.org/hq/index.php?option=com_content&view=article&id=9476:yellow-fever&Itemid=0&lang=en#gsc.tab=0 (2015).

[8] WHO. *Yellow Fever—African Region (AFRO)*. https://www.who.int/emergencies/disease-outbreak-news/item/2022-DON405 (2022).

[9] Lancet T (2018). Yellow fever: A major threat to public health. The Lancet.

[10] WHO. *Fractional Dose Yellow Fever Vaccine as a Dose-Sparing Option for Outbreak Response*. https://www.who.int/publications/i/item/WHO-YF-SAGE-16-1 (2016).

[11] Casey RM (2019). Immunogenicity of fractional-dose vaccine during a yellow fever outbreak—Final report. N. Engl. J. Med..

[12] Simões M (2023). Standardization, validation, and comparative evaluation of a faster and high-performance test for quantification of yellow fever neutralizing antibodies. J. Immunol. Methods.

[13] OPAS/OMS. *Febre Amarela*. https://www.paho.org/pt/topicos/febre-amarela (2024).

[14] PAHO/WHO. *Brazil Launches World’s Largest Campaign with Fractional-Dose Yellow Fever Vaccine*. https://www3.paho.org/hq/index.php?option=com_content&view=article&id=14065:brazil-launches-worlds-largest-campaign-with-fractional-dose-yellow-fever-vaccine&Itemid=0&lang=en#gsc.tab=0 (2018).

[15] WHO. *Yellow Fever Vaccine: WHO Position on the Use of Fractional Doses—June 2017*. https://www.who.int/publications/i/item/who-wer9225 (2017). 10.1016/j.vaccine.2017.06.087 28689653

[16] Campi-Azevedo AC (2014). Subdoses of 17DD yellow fever vaccine elicit equivalent virological/immunological kinetics timeline. BMC Infect. Dis..

[17] Martins RM (2013). 17DD yellow fever vaccine. Hum. Vaccines Immunotherap..

[18] Collaborative Group for Studies on Yellow Fever Vaccines (2014). Duration of post-vaccination immunity against yellow fever in adults. Vaccine.

[19] Monath TP (2001). Yellow fever: An update. Lancet Infect. Dis..

[20] Roukens AHE, van Halem K, de Visser AW, Visser LG (2018). Long-term protection after fractional-dose yellow fever vaccination: Follow-up study of a randomized, controlled, noninferiority trial. Ann. Intern. Med..

[21] Simões M (2012). Evaluation of accuracy and reliability of the plaque reduction neutralization test (micro-PRNT) in detection of yellow fever virus antibodies. Biologicals.

[22] Vratskikh O (2013). Dissection of antibody specificities induced by yellow fever vaccination. PLoS Pathog..

[23] Campi-Azevedo AC (2012). 17DD and 17D–213/77 yellow fever substrains trigger a balanced cytokine profile in primary vaccinated children. PLoS ONE.

[24] Martins MA (2007). Activation/modulation of adaptive immunity emerges simultaneously after 17DD yellow fever first-time vaccination: Is this the key to prevent severe adverse reactions following immunization?. Clin. Exp. Immunol..

[25] Silva ML (2011). Characterization of main cytokine sources from the innate and adaptive immune responses following primary 17DD yellow fever vaccination in adults. Vaccine.

[26] Costa-Pereira C (2018). Multi-parameter approach to evaluate the timing of memory status after 17DD-YF primary vaccination. PLoS Negl. Trop. Dis..

[27] Reis LR (2022). Exploratory study of humoral and cellular immunity to 17DD yellow fever vaccination in children and adults residents of areas without circulation of yellow fever virus. Vaccine.

[28] Akondy RS (2009). The yellow fever virus vaccine induces a broad and polyfunctional human memory CD8+ T cell response. J. Immunol..

[29] Campi-Azevedo AC (2019). 17DD yellow fever revaccination and heightened long-term immunity in populations of disease-endemic areas, Brazil. Emerg. Infect. Dis..

[30] Campi-Azevedo AC (2017). Heparin removal by ecteola-cellulose pre-treatment enables the use of plasma samples for accurate measurement of anti-Yellow fever virus neutralizing antibodies. J. Immunol. Methods.

[31] Lin D, Gupta S, Maecker HT (2015). Intracellular cytokine staining on PBMCs using CyTOF™ mass cytometry. Bio Protoc..

[32] Roukens AH (2008). Intradermally administered yellow fever vaccine at reduced dose induces a protective immune response: A randomized controlled non-inferiority trial. PLoS ONE.

