# Supplementary material for: Comprehensive landscape of neutralizing antibody and cell-mediated response elicited by the 1/5 fractional dose of 17DD-YF primary vaccination in adults
Source: Sci Rep. 2024 Apr 2;14:7709. doi: 10.1038/s41598-024-57645-3 (PMC10987530; doi:10.1038/s41598-024-57645-3)
Supplement: Supplementary file 1 — Supplementary Figures. [file 41598_2024_57645_MOESM1_ESM.pdf]

Comprehensive landscape of neutralizing antibody and cell-mediated response elicited by the 1/5 fractional dose of 17DD-YF primary vaccination in adults

Laise Rodrigues Reis; Ismael Artur Costa-Rocha; Ana Carolina Campi-Azevedo; Vanessa Peruhype-Magalhães; Márcio Sobreira Silva Araújo; Elaine Spezialli; Rosiane Aparecida da Silva-Pereira; Lis Ribeiro do Valle Antonelli; Thais Abdala-Torres; Gregório Guilherme Almeida; Eder Gatti Fernandes; Francieli Fontana Sutile Tardetti Fantinato; Carla Magda Allan Santos Domingues; Maria Cristina Ferreira Lemos; Alexandre Chieppe; Jandira Aparecida Campos Lemos; Jordana Grazziela Coelho-dos-Reis; Sheila Maria Barbosa de Lima; Adriana de Souza Azevedo; Waleska Dias Schwarcz; Luiz Antônio Bastos Camacho; Maria de Lourdes de Sousa Maia; Tatiana Guimarães de Noronha; Caroline Duault; Yael Rosenberg-Hasson; Andréa Teixeira-Carvalho; Holden Terry Maecker*; Olindo Assis Martins-Filho* and Collaborative Group for Studies of Yellow Fever Vaccine

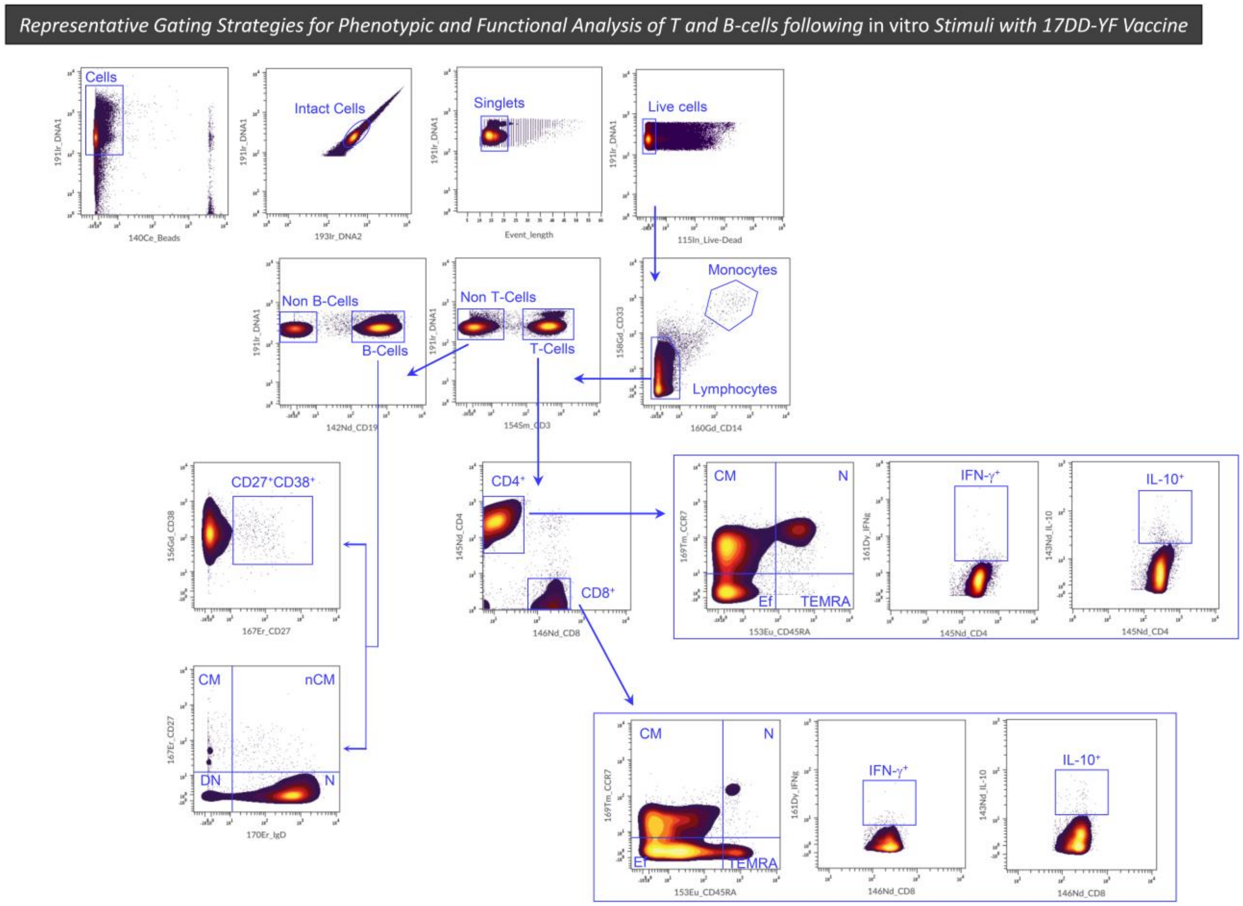

Supplementary Figure 1. Representative gating strategies for phenotypic and functional analysis of T and B-cells following in vitro *stimuli with 17DD-YF vaccine.* Ungated events were sequentially analyzed using Cytobank shared cloud-based platform using a set of gating strategies as follows: i) 140Ce beads *vs* 191Ir DNA1 gating to select cells; ii) 193Ir DNA2 *vs* 191Ir DNA1 plot was used to select singlets identified as positive cells for DNA markers; iii) intact singlets were identified on Event length vs 193Ir DNA2 plot; iv) live intact singlets were gated as negative events for 115In live-dead cell marker; v) lymphocytes were selected as negative events on 160Gd CD14 *vs* 158Gd CD33 plot; vi) T-cells were gated as 154Sm CD3 positive cells and B-cells selected amongst non-T-cells as 142Nd CD19 positive cells; vii) additional phenotypic/functional features were assessed within gated B and T-cells as illustrated by 167Er CD27 *vs* 156Gd CD38

and 170Er IgD *vs* 167Er CD27 for B-cells and 153Eu CD45RA *vs* 169Tm CCR7 and 161Dy IFN-γ or I43Nd IL-10 *vs* 145Nd CD4 and 146Nd CD8 for T-cell subsets.

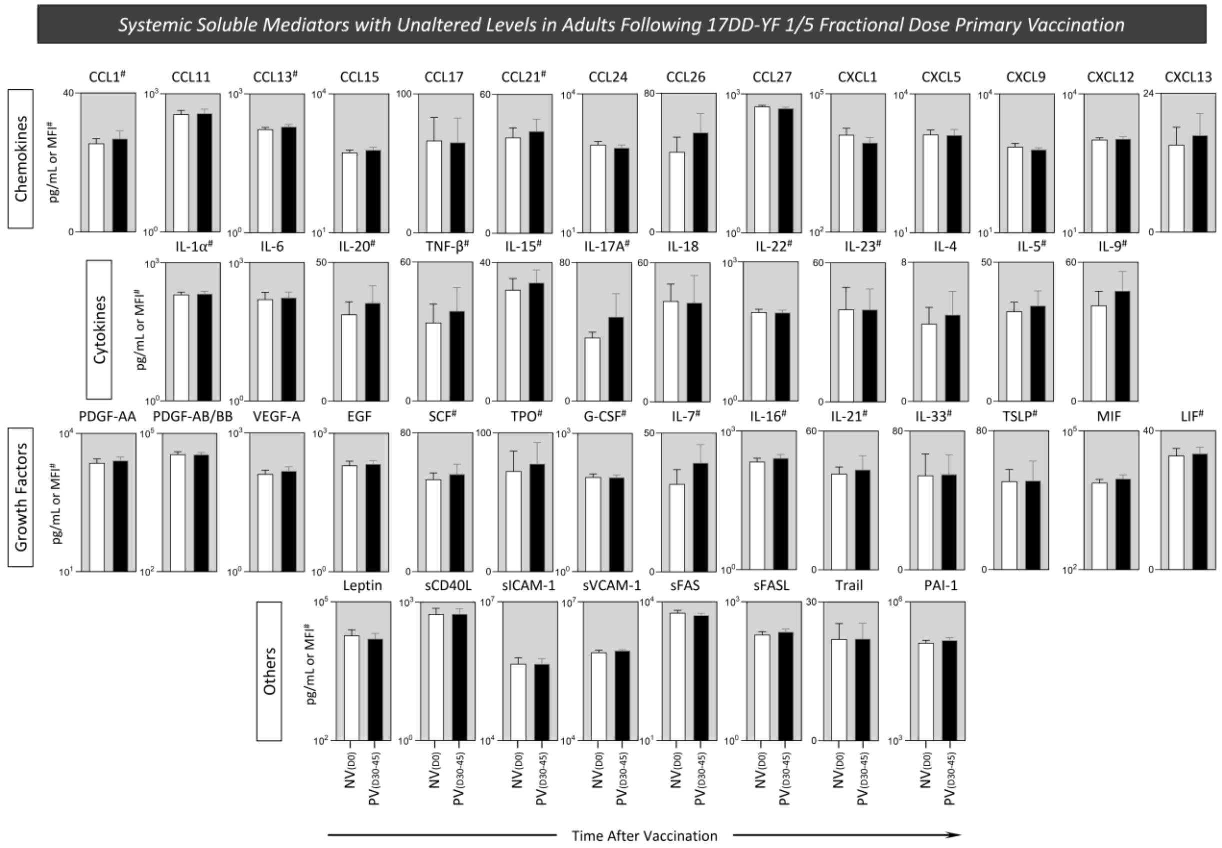

Supplementary Figure 2. Systemic soluble mediators with unaltered levels in adults following 17DD-YF 1/5 fractional dose primary vaccination. Systemic soluble mediators were measured in plasma samples from all volunteers before (NV$_{(D0)}$ = □, n=15) and 30-45 days after (PV$_{(D30-45)}$ = ■, n=15) 17DD-YF 1/5 fractional dose primary vaccination. Measurements were carried out by xMAP technology as described in Materials and Methods. The results are shown as mean values ± standard error of plasma concentration expressed in pg/mL or MFI (#). Comparative analysis between NV$_{(D0)}$ and PV$_{(D30-45)}$ was carried out by Student t test. No significant differences were observed between groups at $p<0.05$. Gray background underscores unaltered values observed at PV$_{(D30-45)}$ as compared to NV$_{(D0)}$.
